# In vitro drug sensitivity and clinical efficacy of the HAG regimen in pediatric AML

**DOI:** 10.1093/oncolo/oyaf340

**Published:** 2025-11-20

**Authors:** Diying Shen, Weiling Yan, Tian Xia, Liping Shang, Jingying Zhang

**Affiliations:** Department of Hematology and Oncology, Pediatric Hematology-Oncology Center, The Children’s Hospital, Zhejiang University School of Medicine, National Clinical Research Center for Child Health, Hangzhou 310052, China; Department of Hematology and Oncology, Pediatric Hematology-Oncology Center, The Children’s Hospital, Zhejiang University School of Medicine, National Clinical Research Center for Child Health, Hangzhou 310052, China; Department of Hematology and Oncology, Pediatric Hematology-Oncology Center, The Children’s Hospital, Zhejiang University School of Medicine, National Clinical Research Center for Child Health, Hangzhou 310052, China; Department of Hematology and Oncology, Pediatric Hematology-Oncology Center, The Children’s Hospital, Zhejiang University School of Medicine, National Clinical Research Center for Child Health, Hangzhou 310052, China; Department of Hematology and Oncology, Pediatric Hematology-Oncology Center, The Children’s Hospital, Zhejiang University School of Medicine, National Clinical Research Center for Child Health, Hangzhou 310052, China

**Keywords:** pediatric, AML, HAG, drug sensitivity, complete remission

## Abstract

**Background:**

Standard induction chemotherapy for acute myeloid leukemia (AML) using cytarabine combined with anthracyclines (epirubicin/IA or daunorubicin/DA) is effective but associated with severe adverse effects. The HAG regimen (low-dose homoharringtonine, cytarabine, G-CSF), known for lower toxicity, shows promise in adult AML, but its application in pediatric AML remains underexplored.

**Methods:**

This study investigated the feasibility and efficacy of the HAG regimen in pediatric AML. Twenty-four newly diagnosed pediatric AML patients (excluding M3 subtype) were enrolled between August 2021 and November 2022. Patients received the HAG regimen as induction therapy. Drug sensitivity testing was performed to evaluate in vitro efficacy.

**Results:**

After the first induction cycle with HAG, the overall complete remission (CR) rate was 75% (18/24). Patients classified as ELN favorable and intermediate-risk achieved a 100% CR rate. The 1-year overall survival (OS) rate was 83.3% ± 7.6%, and the 3-year OS rate was 75.0% ± 8.8%. The 1-year event-free survival (EFS) rate was 75.0% ± 8.8%, and the 3-year EFS rate was 66.1% ± 9.8%.

**Conclusion:**

The HAG regimen demonstrates high efficacy and holds great promise for pediatric AML. It achieves survival outcomes comparable to more intensive regimens while offering the significant advantage of reduced toxicity.

Implications for practice This study demonstrates that the HAG regimen is a highly effective and feasible induction therapy for newly diagnosed pediatric AML (non-M3), achieving promising complete remission rates (75% overall, 100% in ELN favorable/intermediate-risk) and survival outcomes (1-year OS 83.3%, 3-year OS 75.0%) comparable to intensive anthracycline-based regimens. Crucially, HAG offers the significant practical advantage of reduced toxicity. This makes it a valuable alternative treatment option, particularly for patients who may be less tolerant of the severe adverse effects associated with standard induction chemotherapy (eg, IA or DA). These findings strongly support the further investigation of HAG in larger, randomized controlled trials as a potentially less toxic yet equally effective frontline therapy for pediatric AML.

## Background

The standard induction chemotherapy regimen for acute myeloid leukemia (AML), combining cytarabine with either epirubicin (IA regimen) or daunorubicin (DA regimen), is widely regarded as the most effective initial treatment for improving outcomes and prognosis in pediatric patients.^1^^,^^2^ Standard IA/DA regimens use daunorubicin (60-90 mg/m^2^) or idarubicin (12 mg/m^2^) with cytarabine (100-200 mg/m^2^ daily). However, such highly intensive treatment inevitably leads to severe adverse effects, including myelosuppression, organ dysfunction, and other complications. Is there any other more moderate chemotherapy regimen that can achieve better therapeutic efficacy and prognosis?

Low-dose cytarabine is a subcutaneous administration regimen, with a dose significantly lower than the standard intensive regimen (usually ≤20 mg/m^2^ per dose), administered twice daily for 10 to 14 consecutive days as one course of treatment. In 1995, Yamada and colleagues first proposed a novel low-dose chemotherapy regimen for the treatment of AML, consisting of low-dose cytarabine and aclarubicin combined with granulocyte colony-stimulating factor (G-CSF) priming, referred to as the CAG regimen.^3^ Over the past 2 decades, the CAG regimen has been widely used in China and Japan and has proven to be efficacious in the treatment of refractory and relapsed AML as well as high-risk myelodysplastic syndrome (MDS).^4^ However, repeated administrations of anthracyclines can result in dose-dependent cardiac myocytes and interstitial injuries. These injuries are associated with early diastolic and later systolic cardiac dysfunction. Moreover, delayed left ventricular dysfunction may occur several years after therapy has been completed. In a study by Lipshultz et al.,^5^ of 115 children developed overt heart failure within 11 years after the completion of therapy, and 57% presented with impaired left ventricular function on echocardiographic examination.^5^

Homoharringtonine (HHT) was first applied in clinical practice in China and has since been widely used in the treatment of AML. In addition to its definite efficacy, HHT has been shown to have milder toxicity and myelosuppression compared with similar chemotherapy drugs. When HHT was administered at a dose of 5-6 mg/m^2^ daily using a short infusion schedule, cardiovascular failure occurred in 25% of patients.^6^ However, when HHT was continuously infused at a dose of 2.5 mg/m^2^/d for 14 days, the toxicity was minimal.^7^ Therefore, the combination of low-dose HHT, cytarabine, and G-CSF, referred to as the HAG regimen, provides an alternative option that has been proven to be effective in the treatment of in adults in China.^8^^,^^9^ This regimen offers a less toxic alternative for effectively controlling the disease in this patient population. Compared with the historical control group receiving standard-dose chemotherapy, patients treated with the HAG regimen have higher overall response rates and complete response rates. Moreover, the HAG regimen is well-tolerated, with a low incidence of serious adverse events.

There are few relevant research reports on the use of HAG in childhood AML. This study aims to explore the sensitivity of HAG regimen to childhood AML cells in vitro and in vivo, in order to evaluate its efficacy and potential.

## Materials and methods

### Patients and inclusion criteria

A total of 24 newly diagnosed AML patients (excluding M3 subtype) admitted to our department between August 2021 and November 2022 were enrolled in this study. All patients received low-intensity chemotherapy with the HAG regimen. These patients were treated with low-intensity chemotherapy HAG. Clinical samples for drug sensitivity testing were collected prior to treatment initiation. Gene mutations, chromosomes, and other samples of the included patients were collected before treatment. Clinical samples for evaluating the efficacy were collected from the 21st day to the 28th day after the end of chemotherapy. All patients provided written informed consent. The study was approved by the Human Ethics Committee of the Children’s Hospital, Zhejiang University School of Medicine (Ethical code: 2025-IRB-0090-P-01) and complied with the ethical guidelines of the Helsinki Declaration, revised in 1975. The inclusion criteria were as follows: (1) Newly diagnosed patients: leukemia type and risk level were categorized according to the 2016 WHO classification and the 2017 European Leukaemia Net (ELN) risk stratification.^10^^,^^11^ (2) Age ≤ 18 years at diagnosis. (3) About to receive HAG chemotherapy as induction.

### Treatment

The HAG treatment regimen was administered as follows: HHT was administered via intravenous infusion at a dose of 2 mg/m^2^/day from day 1 to day 14; cytarabine was administered via subcutaneous injection at a dose of 10 mg/m^2^ every 12 hours from day 1 to day 14 and G-CSF was administered via sub­cutaneous injection at a dose of 200 μg/m^2^/day from day 1 to day 14 (with a maximum dose of 300 μg). Following the achievement of complete remission (CR) with induction therapy, the majority of patients received 4 to 6 cycles of high-dose cytarabine as consolidation therapy. To date, 11 patient received allogeneic hematopoietic stem cell transplantation (Allo-HSCT) after CR. For patients who failed to respond to the HAG regimen, salvage therapies, such as MA (mitoxantrone plus cytarabine), FLAG (fludarabine, cytarabine, and G-CSF), or decitabine-based combination therapies were administered.

### Response criteria and endpoint

CR was defined by the presence of normal cellular bone marrow with fewer than 5% blasts, along with a neutrophil count ≥1.0 × 10^9^/L and a platelet count ≥100 × 10^9^/L in peripheral blood, and transfusion-independent status. Overall survival (OS) was defined as the time interval from the initial diagnosis to death, or censored at the last follow-up date if the patient was still alive. Toxic effects were graded according to the ­Common Terminology Criteria for Adverse Events (CTCAE) Version 5.0.

### High-throughput drug sensitivity screening

Bone marrow was collected under aseptic conditions, placed in an EDTA anticoagulant tube, refrigerated at 2 -8°C, and transported to PreceDo Inc. Primary tumor cells were isolated and purified according to standard operating procedures and expanded in vitro using an improved cell reprogramming technique. The cultured primary cells were tested for high-throughput drugs in vitro according to the clinical first-line and second-line treatment schemes of the corresponding cancer types and FDA drug bank, and the sensitive drugs and schemes were selected. The experiment was conducted following the procedure described in a previous report.^12^^,^^13^ To evaluate drug susceptibility, cells in the logarithmic growth phase were harvested and plated in white 384-well culture plates at a density of 1 × 10^5^ cells/mL. Each well received 50 μL of cell suspension. Pharmaceutical compounds were dissolved and serially diluted in dimethylsulfoxide (DMSO), with concentrations calibrated to clinically relevant levels according to international standards.^14^ The control group was treated with DMSO. Drug (0.1 μL) was added to each plate using a JANUS automated workstation (Perkin Elmer Inc.). Following 72-hour incubation, cellular viability was quantified by adding 10 μL CellTiter-Glo reagent per well. Fluorescence was measured using an EnVision plate reader (Perkin Elmer Inc.). The growth inhibition rates of ­different chemotherapeutic drugs were calculated in the laboratory, and test reports were prepared in the clinic. In contrast to the reference, drug inhibition rates were categorized as ­follows: high sensitivity (+++; inhibition rate ≥80%), moderate sensitivity (++; inhibition rate 50%-79%), and low sensitivity (+; inhibition rate 20%-49%).

### Data analysis

Statistical analysis was performed using SPSS version 10.0 (IBM Corp.). Fisher’s exact test was used to identify factors influencing the CR rate. All reported *P* values are 2-sided, and a *P* value < .05 was considered statistically significant. The survival rates for event-free survival (EFS) and OS were estimated using the Kaplan-Meier method.

## Results

### Patient characteristics

**Figure 1.**
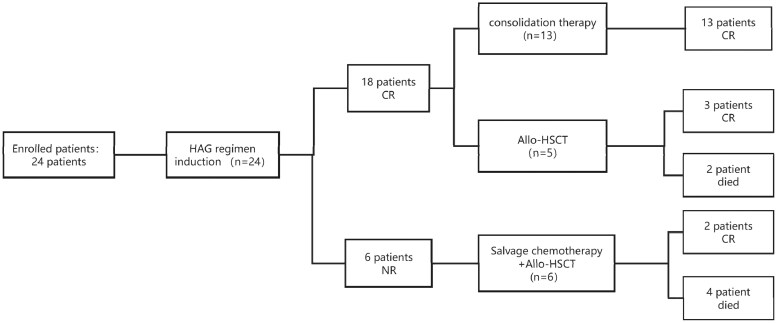
Patient enrollment, allocation, treatment, and outcomes.

A total of 24 newly diagnosed AML patients were enrolled in our study. The cohort included 11 females and 13 males, with 22 patients having primary AML and 2 having therapy-related AML. Additionally, 14 patients were under 10 years old, and 10 were aged 10 years or older. The median age of the patients was 7.6 years (range 0.5-13.91 years). All patients underwent genetic assessment prior to treatment. It was found that 62.5% (15/24) were at adverse risk according to ELN stratification (Table 1). In the group, there are 4 cases with the *NUP98::NSD1* gene, 3 of which also have the *FLT3::ITD* gene. One case of therapy-related leukemia is associated with the *KAT6A::CREBBP* gene, while another case of therapy-related leukemia is associated with a chromosomal abnormality of t (3; 3) (q21; q26.2) and −7. Only one patient has intermediate risk with an *MLLT3::KMT2A* gene, and 8 patients are at favorable risk, among which 5 have the *RUNX1::RUNX1T1*. A corsort flow diagram details patient enrollment, allocation, treatment, and outcomes (Figure 1).

**Table 1.** Genetic characteristics of patients.

Risk category	Genetic abnormality	No.	
Favorable	t(8; 21)(q22; q22.1); *RUNX1::RUNX1T1*	5	8
	Mutated *NPM1*, without *FLT3::ITD*	1	
	*bZIP* in-frame mutated *CEBPA*	2	
Intermediate	t(9; 11)(p21.3; q23.3); *MLLT3::KMT2A*	1	1
Adverse	t(6; 9)(p23; q34.1); *DEK::NUP214*	1	15
	t(v; 11q23.3); *KMT2A* rearranged	3	
	inv(3)(q21.3q26.2) or t(3; 3)(q21.3; q26.2); *GATA2*; *MECOM* (*EVI1*)	3	
	t(8; 16)(p11; p13)/*KAT6A::CREBBP*	1	
	11p15/*NUP98* rearranged	4	
	Complex karyotype	1	
	monosomal karyotype	1	
	Mutated *ASXL1*	1	

### Drug sensitivity test results

Single-agent sensitivity analysis was performed on all 24 patients using cytarabine (Ara-C) and HHT (Table 2). For cytarabine monotherapy sensitivity analysis: in the 100 mg/m^2^ group, no cases showed high sensitivity, and 7/24 (29.2%) exhibited moderate sensitivity. Higher sensitivity rates were observed in the high-dose groups. Specifically, the 2000 mg/m^2^ group had no cases of high sensitivity and 22/24 (91.7%) with moderate sensitivity, while the 3000 mg/m^2^ group included 1/24 (4.2%) case of high sensitivity and 22/24 (91.7%) with moderate sensitivity. For HHT monotherapy sensitivity analysis, the 1 mg/m^2^ group had 1/24 (4.2%) case of high sensitivity and 15/24 (62.5%) with moderate sensitivity. The 2 mg/m^2^ group showed 1/24 (4.2%) case of high sensitivity and 17/24 (70.8%) with moderate sensitivity.

**Table 2.** Drug sensitivity results for each patient.

Drug			High sensitivity	Moderate sensitivity	Low sensitivity
Ara-c		100 mg/m^2^	0% (0)	29.2% (7)	70.8% (17)
		2000 mg/m^2^	0% (0)	91.6% (22)	8.4% (2)
		3000 mg/m^2^	4.2% (1)	91.6% (22)	4.2% (1)
HHT		1 mg/m^2^	4.2% (1)	62.5% (15)	33.3% (8)
		2 mg/m^2^	4.2% (1)	70.8% (17)	25.0 (6)
HA	HHT	2 mg/m^2^	33.3% (8)	66.6% (16)	0 (0%)
	Ara-c	10 mg/m^2^			
DA	daunorubicin	40 mg/m^2^	29.1% (7)	62.5% (15)	8.3% (2)
	Ara-c	200 mg/m^2^			
IA	idarubicin	12 mg/m^2^	12.5% (3)	75.0% (18)	12.5% (3)
	Ara-c	100 mg/m^2^			

The combined drug sensitivity of HHT (2000 mg/m^2^) and cytarabine (10 mg/m^2^) was also evaluated. All patients exhibited sensitivity to the combination, with 8 patients (8/24, 33.3%) showing high sensitivity and 16 patients (16/24, 66.7%) demonstrating moderate sensitivity. The sensitivity rate of the HA regimen was found to be non-inferior to that of traditional chemotherapy regimens, IA and DA (*P = *.104).

### Response evaluation

After the first induction treatment cycle of HAG, the total CR rate was 75% (18/24). An encouragi*n*g CR rate of 100% was achieved in the ELN favorable and intermediate-risk groups with HAG treatment. In the adverse-risk population, the CR rate was 60% (9/15). Among the 6 children who did not achieve remission, all had adverse risk. Specifically, all 3 children with *NUP98::NSD1* and *FLT3::ITD* failed to respond to treatment. The remaining unremitted children included one with *DEK::NUP214*, one with secondary leukemia associated with *KAT6A::CREBBP*, and another with *AXSL1* and *SRSF2*. Among them, only one patient who did not achieve remission showed high sensitivity in the drug sensitivity test. Our correlation analysis between in vitro drug sensitivity and clinical response to HAG therapy revealed distinct patterns. While no significant associations emerged for cytarabine monotherapy (Ara-C mono), HHT monotherapy sensitivity (HHT mono, 1 mg/m^2^ group) or the HHT-cytarabine combination (HHT+Ara-C combo) with remission rates (*P *> .05), HHT monotherapy sensitivity (HHT mono, 2 mg/m^2^ group) demonstrated statistically significant correlations with clinical outcomes (*P = *.006).

### Survival

**Figure 2.**
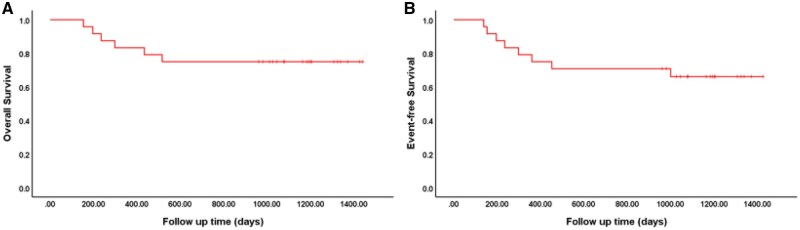
Overall survival (OS) and event-free survival (EFS) of AML patients. The median OS and EFS were not reached. The 1-year OS was 83.3% ± 7.6%, and the 3-year OS was 75.0% ± 8.8%. The 1-year EFS was 75.0% ± 8.8%, and the 3-year EFS was 66.1% ± 9.8%.

The median follow-up for the entire cohort was 647 days (range, 153-1017). The median OS and EFS were not reached. Eleven patients (45.8%) underwent HSCT. Patients treated with HAG achieved a 1-year OS of 83.3% ± 7.6% and a 3-year OS of 75.0% ± 8.8%, as well as a 1-year EFS of 75.0% ± 8.8% and a 3-year EFS of 66.1% ± 9.8% (Figure 2). We analyzed the clinical outcomes of patients who underwent HSCT. No statistically significant differences in OS or EFS were observed between the HSCT group and the non-HSCT group (*P *> .05). However, due to the limitations of this study’s small sample size and potential biases, these findings cannot be considered definitive conclusions regarding the efficacy of HSCT. Future larger-scale prospective studies or matched cohort analyses will be critical to clarify the role of HSCT in this patient population.

### Analysis of factors influencing CR in AML with HAG treat

There were no statistically significant differences in the CR rate based on age, gender, or white blood cell counts (*P *= .341, *P *= 1.000, and *P *= .127) (Table 3). Gene mutations are common in AML, and mutations in genes such as *NUP98::NSD1*, *KMT2A* rearranged, and *FLT3::ITD* contribute to the development of AML. These mutations are often associated with poor prognosis. In this study, we found that among 4 patients with *NUP98::NSD1* mutations, the CR rate was 25%. The 3 patients who did not achieve remission all had low mutant allelic ratio (MAR) of *FLT3-ITD* (MAR < 0.5). All 8 patients with favorable factors achieved remission, with 7 of them not undergoing transplantation and surviving long-term. Only one patient who underwent transplantation died due to graft-versus-host disease.

**Table 3.** Response to treatment modalities.

	CR	NR	*P*-value
Age			.341
<10 years	9 (64.5%)	5 (35.7%)	
≥10 years	9 (90.0%)	1 (10.0%)	
Gender			1.000
M	10 (76.9%)	3 (23.1%)	
F	8 (72.7%)	3 (27.3%)	
WBC			.127
<100			
≥100			
Genetic anomaly at diagnosis			.002
*RUNX1::RUNX1T1*	5	0	
*NPM1*	1	0	
*bZIP* in-frame mutated *CEBPA*	2	0	
*KMT2A* rearranged	4	0	
*GATA2*	2	0	
*NUP98::NSD1* with *FLT3::ITD*	0	3	
*NUP98::NSD1*	1	0	
*ASXL1*	0	1	
*KAT6A::CREBBP*	0	1	
*DEK::NUP214*	0	1	
t(3; 3)(q21.3; q26.2)	1	0	

### Serious adverse events

Overall, the HAG treatment regimen demonstrated a manageable safety profile. As detailed in Table 4, the most frequent adverse events observed during induction therapy predominantly comprised hematological toxicities. Grade 3 and 4 toxicities were primarily thrombocytopenia and neutropenia, with a notably low incidence of non-hematological events. Myelosuppression was universal, and febrile neutropenia occurred in 75.0% (18/24) of patients; however, only 33.3% (8/24) experienced grade 3-4 febrile neutropenia. Critically, no induction-related deaths occurred. Among patients achieving CR, the median time to granulocyte recovery (absolute neutrophil count ≥ 1.0 × 10^9^/L) was 11 days (range, 2-24 days), while platelet recovery (platelet count ≥ 50 × 10^9^/L) required a median of 17 days (range, 2-24 days).

**Table 4.** Most frequent side effects during induction therapy.

Side effect	Grade I/II No. of patients (%)	Grades III/IV No. of patients (%)
Neutropenia	3 (12.5%)	21 (87.5%)
Thrombocytopenia	1 (4.2%)	24 (95.8%)
Febrile neutropenia	10 (41.6%)	8 (33.3%)
Nausea, vomiting	6 (25%)	0 (0%)
Increased serum ALT or AST	9 (37.5%)	0 (0%)
Creatinine elevation	0 (0%)	0 (0%)
Cardiac dysfunction	0 (0%)	0 (0%)

## Discussion

HHT is a plant alkaloid first isolated from *Cephalotaxus* in China and has been used successfully in the treatment of acute and chronic myeloid leukemia since the 1970s.^15^^,^^16^ HHT is a protein synthesis inhibitor that causes leukemic cells to arrest at the G1/G2 phase of the cell cycle^17^^,^^18^ and can induce apoptosis in leukemia cells by interfering with the function of ribosomes and controlling DNA synthesis.^17^^,^^19^^,^^20^ Cytarabine acts at the S-phase of the cell cycle to induce apoptosis. G-CSF enriches S-phase leukemic blasts,^21^ thereby enhancing the efficacy of cytarabine, which is active during the S-phase.

In this study, we investigated the in vivo and in vitro sensitivity of the HAG regimen in 24 children newly diagnosed with AML. Drug sensitivity testing revealed that all patients showed sensitivity in vitro to the combined HHT and cytarabine ­regimen, with a significant proportion demonstrating high ­sensitivity. This aligns with the non-inferiority of HAG’s sensitivity rate compared to traditional chemotherapy regimens IA and DA (*P = *.104), suggesting that HAG could be a viable alternative.

Previous studies have reported a CR rate of 55% (95% CI, 46%-63%) in patients with AML or myelodysplastic syndrome (MDS) treated with the HAG regimen during induction therapy, with the CR rate reaching 60% in elderly AML patients.^22^ These results are encouraging and suggest that the HAG regimen may be effective in pediatric AML, particularly in low- and intermediate-risk populations. In our study, a 75% CR rate was observed after the first induction treatment cycle with HAG, including a 100% CR rate in the ELN favorable and intermediate-risk group. Correlation analysis between *in vitro* drug sensitivity testing and clinical outcomes indicates that HHT sensitivity testing may serve as a predictive biomarker for the efficacy of the HAG regimen. HHT-sensitive patients (2 mg/m^2^) represent strong candidates for HAG therapy, whereas HHT-resistant subgroups—particularly those with adverse-risk disease—demonstrate reduced response rates. For these individuals, tailored therapeutic alternatives should be selected based on individualized risk-benefit assessments.

In terms of genetic mutations, the study found that among patients with *NUP98::NSD1* mutations, the CR rate was only 25%. Notably, all 3 patients who did not achieve remission also exhibited co-expression of the *FLT3::ITD* gene. Rearrangements of the *NUP98* gene are associated with high malignancy in pediatric AML, characterized by poor initial responses to conventional chemotherapy and suboptimal treatment outcomes.^23^  *NUP98-NSD1*-positive AML often coexists with additional mutations in genes such as *NRAS*, *FLT3*, *WT1*, and *MYC*.^24^ When *NUP98::NSD1* and *FLT3* coexist in pediatric AML patients, the malignancy of the disease may further increase, and the therapeutic response could be even worse.^25^ Previous literature reports indicate that patients with dual genetic alterations (*NUP98::NSD1* and *FLT3::ITD*) exhibit a poor response to conventional chemotherapy, with an induction remission rate of less than 30%. Our results are also generally consistent with these findings. This highlights the importance of genetic profiling in predicting treatment response and underscores the potential need for personalized treatment strategies, especially for patients with specific genetic abnormalities that confer a poor prognosis.

Only a few studies have reported survival data for patients treated with the HAG regimen. Among them, a study by Chen and colleagues reported that the median OS for newly diagnosed AML patients treated with HAG was 12.0 ± 1.7 months.^26^ In our study, although the median OS and EFS were not reached, the survival data are promising. Specifically, the 1-year and 3-year OS rates were 83.3% and 75.0%, respectively, while the EFS rates were 75.0% and 66.1%, respectively. It is important to note that these survival outcomes may have been influenced by subsequent consolidation therapy received by all patients, including high-dose cytarabine and HSCT where indicated.

Our findings indicate that the HAG regimen demonstrates a favorable safety profile with low treatment-related toxicity. The observed overall CR rate was clinically meaningful, complemented by encouraging 3-year OS and EFS outcomes. Particularly noteworthy is the high CR rate achieved in low- and intermediate-risk patients, which may correlate with sustained survival advantages in these subgroups. Nevertheless, this study has inherent limitations, including its modest sample size and absence of randomized comparison against standard chemotherapy backbones. These findings warrant further validation through large-scale randomized controlled trials to establish the regimen’s definitive therapeutic position.

## Conclusion

The low-dose regimen of HAG represents a significant advancement in the treatment of pediatric AML, offering a less toxic and effective alternative to traditional intensive chemotherapy. Its high response rates, favorable survival outcomes, and reduced toxicity make it a promising candidate for frontline therapy. However, the importance of genetic profiling and the potential integration of targeted therapies highlight the need for personalized treatment strategies. Future research should focus on optimizing HAG and exploring its role in combination with emerging therapies to further improve outcomes for pediatric AML patients.

## Data Availability

The clinical data underlying this study are subject to strict patient-privacy regulations and cannot be made publicly available in their raw form. However, de-identified, aggregated datasets that support the main findings are available upon reasonable request to the corresponding author (contact details provided in the manuscript). All requests will be reviewed by the institutional ethics committee to ensure compliance with local privacy laws before any data are shared.
